# Recreational Use of Efavirenz by Young People Living With HIV in Zimbabwe

**DOI:** 10.1177/23259582251370550

**Published:** 2025-08-19

**Authors:** Rhulani Beji-Chauke, MSc, Kudzai Hlahla, MSc, Katya Govender, PhD, Rashida A Ferrand, PhD, Victoria Simms, PhD

**Affiliations:** The Health Research Unit Zimbabwe, 4616 Biomedical Research and Training Institute, Harare, Zimbabwe; The Health Research Unit Zimbabwe, 4616 Biomedical Research and Training Institute, Harare, Zimbabwe; 145749 Africa Health Research Institute, Durban, South Africa; The Health Research Unit Zimbabwe, 4616 Biomedical Research and Training Institute, Harare, Zimbabwe; Clinical Research Department, 4906 London School of Hygiene & Tropical Medicine, London, UK; The Health Research Unit Zimbabwe, 4616 Biomedical Research and Training Institute, Harare, Zimbabwe; International Statistics and Epidemiology Group, 4906 London School of Hygiene & Tropical Medicine, London, UK

**Keywords:** Antiretroviral Therapy (ART), Non-Nucleoside Reverse Transcriptase Inhibitor (NNRTI) < Antiretroviral Therapy (ART), Substance use disorders < Psychosocial Aspects of HIV/AIDS, Vulnerable Populations < Healthcare Access and Disparities, Epidemiology of HIV/AIDS

Dear Editor,

During a prevalence survey of young people conducted between October 2022 and March 2023 in Zimbabwe, we collected dried blood spots (DBS) from youth aged 18-24 within 24 clusters in 3 provinces and tested them for HIV and viral load.^1^ Participants who self-reported as HIV negative or not knowing their status, but whose HIV antibody test was positive and whose viral load was under 10,000 copies/ml, were further tested for the presence of antiretroviral drugs (ARVs) in the same sample to determine whether they were in fact receiving treatment. Out of 17,682 survey participants, 791 tested HIV-positive without self-reporting as HIV-positive, and 497/791 had a viral load <10,000 copies. Of these, 486 survey participants had ARV tests on their DBS sample. We tested for efavirenz, tenofovir, atazanavir, ritonavir, nevirapine, abacavir, lamivudine and dolutegravir. We used chi-square tests to investigate the association between efavirenz detection and exposure variables.

Efavirenz was detected in 99 (20.4%) of samples from youth who did not report being HIV-positive and had a viral load <10,000 copies (Table 1). In 70 (70.7%) of these, no other ARV was detected. Detection of efavirenz was particularly common in 3 of the 24 clusters surveyed (Glenview, Budiriro, Hopley), while 12 clusters had no cases at all. Efavirenz detection was more common in males (29.4%) than females (14.4%; χ^2^ = 16.2, *P* < .001), more common among those currently in education than not (χ^2^ = 33.2, *P* < .001), and more common among participants with an HIV viral load >1000 copies (defined as unsuppressed by WHO^2^) than those virally suppressed (χ^2^ = 22.3, *P* < .001). By contrast, the other 7 ARVs were associated with female sex and viral suppression. The survey included questions about use of recreational drugs. Only 5.1% of the youth reported any substance use. Substance use was not associated with efavirenz detection (χ^2^ = 0.12, *P* = .73), but self-reported use of prescription drugs for non-medical reasons was associated with efavirenz detection (χ^2^ = 5.6, *P* = .018).

**Table 1.** Efavirenz Detection by Sociodemographic Characteristics among Survey Participants who did not Self-Report as HIV-Positive but Tested HIV-Positive with a Viral Load <10,000 Copies (*N* = 486).

		**Total with *N***	**Efavirenz detected, *n* (%)**	**χ^2^, *P***
	Total	486	99 (20.4)	
**Sex**	Male	194	57 (29.4)	16.2, *P* < .001
	Female	292	42 (14.4)	
**Age**	18-20	201	46 (22.9)	1.3, *P* = .25
	21-24	285	53 (18.6)	
**Viral load**	<1000	255	31 (12.2)	
	1000-9999	231	68 (29.4)	22.3, *P* < .001
**Ever consumed alcohol**	No	387	85 (22.0)	2.6, *P* = .11
	Yes	96	14 (14.6)	
	Missing	3	0	
**Main occupation**	In education	136	50 (36.8)	33.2, *P* < .001
	Employed	106	10 (9.4)	
	Not in education or employed	244	39 (16.0)	
**Other ARVs detected**	Yes	215	29 (13.5)	11.3, *P* < .001
	No	271	70 (25.8)	
**Use prescription drugs**	Yes	10	5 (50.0)	5.6, *P* = .018
	No	473	93 (19.7)	
	Missing	3	1	
**Use any drugs**	Yes	26	6 (23.1)	0.12, *P* = .73
	No	458	93 (20.3)	
	Missing	2	0	
**Cluster**	Glenview, Harare	28	23 (82.1)	231, *P* < .001
	Budiriro, Harare	57	39 (68.4)	
	Hopley, Mashonaland East	40	19 (47.5)	
	Sunningdale, Harare	25	6 (24.0)	
	Pelendaba, Bulawayo	20	4 (20.0)	
	Zengeza, Bulawayo	8	1 (12.5)	
	Mzilikazi, Bulawayo	31	2 (6.5)	
	Emakhandeni, Bulawayo	18	1 (5.6)	
	Dzivarasekwa, Harare	21	1 (4.8)	
	Pumula, Bulawayo	22	1 (4.6)	
	St Mary's, Mashonaland East	27	1 (3.7)	
	Mufakose, Harare	28	1 (3.6)	
	Remaining 12 clusters	161	0	

Efavirenz has psychoactive properties and is an ingredient in the drug cocktail known in South Africa as nyaope or whoonga.^3^ Nyaope and the use of ARVs for recreational purposes in Zimbabwe have been reported in local mainstream media. A survey of 293 people who used drugs in 2022 found that 3/53 (5.7%) in Bulawayo and 1/61 (1.6%) in Harare reported using nyaope in the past year, while 20/53 (37.7%) in Bulawayo and 27/61 (44.3%) in Harare reported using cough syrup, and 4/53 (7.5%) in Bulawayo and 16/61 (26.2%) in Harare reported taking other pharmaceuticals for recreational purposes.^4^ In focus group discussions, participants reported use of ARVs, haloperidol, diazepam and other pharmaceuticals for non-medical reasons. Additionally, focus group participants in Manicaland reported use of nyaope.^4^ In South Africa, 8 out of 43 participants in a qualitative study in Soweto reported recreational use of ARVs broadly, with 1 participant specifically mentioning efavirenz.^5^ In a qualitative study on potential recreational use of HIV medication in Durban, 3 participants reported efavirenz being an active ingredient in whoonga.^6^

We posit that the distribution of efavirenz detection in this survey among those who did not report being HIV-positive is suggestive of recreational use by some participants rather than use of efavirenz as part of antiretroviral therapy. While the 29 participants in whom other ARVs were detected are likely to be diagnosed and on treatment, the evidence suggests that at least some of the 70 in whom only efavirenz was detected were taking it recreationally. These young people may not have known they were HIV-positive. This poses risks of developing resistance to efavirenz and also of interaction with other medications. The clusters where efavirenz detection was most prevalent, Glenview and Budiriro, which border each other, are 2 of the oldest and most high-density neighbourhoods in Harare. Hopley is a new and informal settlement on the outskirts of Harare with reports of high unemployment, low incomes and high levels of sex work and drug use.^7^

The possible recreational use of efavirenz by young people in urban areas is of public health concern, particularly given recent reports of a substantial increase in substance use among youth in Zimbabwe.^8^ Tenofovir/lamivudine/dolutegravir is recommended for first-line therapy in Zimbabwe, with the exception of women who might become pregnant or are in their first trimester, for whom efavirenz is the recommended alternative.^9^ Thus, efavirenz continues to have an important role in HIV treatment in Zimbabwe, while its detection in young men is suggestive of use outside therapy. The use of efavirenz-based cocktails has been shown to have various biopsychosocial consequences and warrants further investigation. Further research is required to determine the epidemiology of recreational efavirenz use in Zimbabwe. This will enable policymakers to develop interventions for harm reduction.
